# Aglycone, Glycoside, or Glucuronide? Experimental and Mechanistic Insights into the Antioxidative Potential of Gossypetin, Gossypin, and Hibifolin

**DOI:** 10.1021/acs.jpcb.5c03338

**Published:** 2025-07-16

**Authors:** Maciej Spiegel, Adam Kowalczyk

**Affiliations:** a Department of Organic Chemistry and Pharmaceutical Technology, Faculty of Pharmacy, 49550 Wroclaw Medical University, Borowska-211A, Wroclaw 50-556, Poland; b Department of Pharmacognosy and Herbal Medicine, Faculty of Pharmacy, 49550 Wroclaw Medical University, Borowska 211A, Wroclaw 50-556, Poland

## Abstract

Oxidative stress, caused by an imbalance between reactive oxygen species and antioxidants, drives chronic diseases such as cancer and neurodegeneration. This study investigates the antioxidant potential of three flavonoids from the *Malvaceae* family—gossypetin, gossypin, and hibifolin—using DPPH (radical scavenging) and FRAP (reducing power) assays, backed by quantum mechanical computations. Gossypetin displayed exceptional scavenging (TEAC: 111.53 mM/g) and reducing power (TEAC: 155.24 mM/g), thanks to its hydroxyl-rich structure, positioning it as a promising therapeutic option for oxidative stress-related conditions. Gossypin provided moderate scavenging (TEAC: 41.68 mM/g) but robust reducing capacity (TEAC: 126.28 mM/g), making it well-suited for food preservation. Hibifolin, with its stable glucuronyl group, showed balanced scavenging and reducing abilities (TEAC: 39.99 mM/g; 94.67 mM/g), ideal for nutraceuticals. Quantum mechanical analyses revealed the mechanisms behind these antioxidant effects, shedding light on their performance.

## Graphical abstract

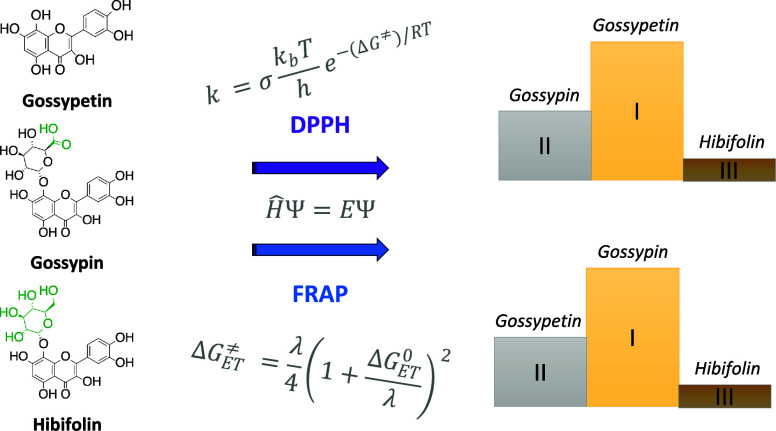

## 1 Introduction

Oxidative stress (OS), defined as an imbalance between the production of reactive oxygen species (ROS) and the body’s antioxidant defenses, underpins the pathogenesis of numerous chronic diseases, including cancer, cardiovascular disorders, and neurodegenerative conditions.^1^ Excessive ROS can damage cellular lipids, proteins, and nucleic acids, thereby disrupting essential biological functions and accelerating degenerative processes. The investigation of effective strategies to counteract OS has shifted focus to natural antioxidants due to the potential toxicity and limited efficacy of synthetic alternatives, such as butylated hydroxytoluene and butylated hydroxyanisole.^2^ Natural antioxidants not only offer improved safety profiles, but also provide additional therapeutic benefits, as demonstrated in recent studies exploring their roles in ameliorating inflammatory and metabolic conditions.^3^

Flavonoids are a diverse class of polyphenolic compounds that are widely distributed in plants and are among the most promising natural antioxidants. These compounds function by neutralizing free radicals, chelating pro–oxidant metals, and modulating oxidative enzyme activity.^4^ Gossypetin (**Gspt**), gossypin (**Gsp**), and hibifolin (**Hbf**) (Figure 1), compounds from the *Malvaceae* family, are notable due to their structural versatility and biological activities.^5^

**Figure 1:**
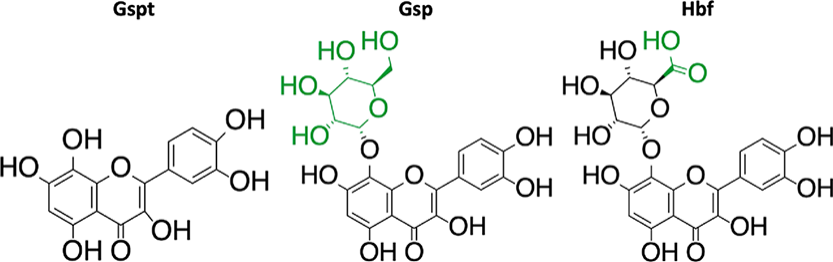
Structures of gossypetin (**Gspt**), gossypin (**Gsp**), and hibifolin (**Hbf**) with green-marked consecutive change in the backbone.

**Gspt**, a 3,3′,4′,5,7,8–Hexahydroxyflavone, demonstrates strong radical scavenging and metal–chelating properties that are essential for counteracting OS–induced cellular damage.^6^ Recent studies have elucidated its potential nephroprotective role through the inhibition of key pathways, such as NF−κB and xanthine oxidase, positioning it as a candidate for managing OS–related kidney injuries.^7^ Furthermore, its dual role in reducing OS and activating AMP–activated protein kinase (AMPK) has revealed potential applications in addressing complex metabolic disorders such as nonalcoholic steatohepatitis (NASH).^6^ **Gspt** capacity to modulate oxidative pathways and reduce inflammation has also been investigated in periodontitis, in which it limits bone resorption and osteoclastogenesis.^8^ **Gspt**, isolated from *Sterculia diversifolia*, demonstrates significant antiglycation and antioxidant activity. Investigations conducted on extracts from the bark and leaves of this plant revealed a 46.98% inhibition of protein glycation, rendering it a promising candidate for further research in the context of diabetes therapy. Its antioxidant properties may confer protection against damage induced by advanced glycation end products (AGEs), which are associated with various diabetes complications. These properties could constitute an important element in the pursuit of natural therapeutic agents for individuals with diabetes.^9^

**Gsp**, a 3,3′,4′,5,7,8–Hexahydroxyflavone 8–glucoside (gossypetin 8–glucoside), exhibits enhanced solubility in aqueous systems, rendering it suitable for applications in food emulsions and beverages. This structural modification enables it to maintain its antioxidant activity in hydrophilic environments, thereby expanding its utility in therapeutic formulations. Its cardioprotective effects against ischemia/reperfusion injuries have been attributed to its capacity to modulate OS, reduce inflammatory cytokine levels, and preserve cardiac function.^10^ Furthermore, **Gsp** has demonstrated the ability to ameliorate nephrotoxicity induced by methotrexate, further underscoring its potential as a therapeutic agent.^7^ Moreover, **Gsp** has exhibited promise in mitigating the pathological effects of lipid peroxidation in pulmonary tissues, highlighting its broad therapeutic potential.^11^ The mechanism of action of **Gsp** as an antioxidant is attributed to its capacity to neutralize free radicals, thereby protecting cells from oxidative stress. This process involves the detection and elimination of reactive oxygen species, which are crucial for preventing cellular damage and the development of oxidation–related diseases. Regarding its anti–inflammatory effects, **Gsp** inhibits signaling pathways associated with inflammatory processes, resulting in a reduction in the expression of pro–inflammatory cytokines, such as TNF−α, IL–1β, and IL–6. This leads to decreased inflammatory cell infiltration and alleviation of inflammation symptoms. **Gsp** also demonstrates analgesic effects, indicating its ability to increase the pain threshold in experimental models. This mechanism may be associated with the modulation of pain pathways in the nervous system, contributing to the reduction in pain sensation. As a neuroprotective agent, **Gsp** safeguards nerve cells from damage, which may be particularly significant in neurodegenerative diseases. It functions by reducing oxidative stress and preventing neuronal apoptosis. **Gsp** exhibits the ability to inhibit the growth of cancer cells and induce apoptosis. These mechanisms may involve effects on the signaling pathways associated with cell proliferation and survival. Furthermore, it demonstrates potential in diabetes management, which may be related to its capacity to enhance insulin sensitivity and regulate blood glucose levels.^12^

**Hbf**, a 3,3′,4′,5,7,8–hexahydroxyflavone 8–glucuronide (gossypetin 8–glucuronide), is characterized by its stable glycosidic bond, which ensures prolonged antioxidant activity and resistance to enzymatic hydrolysis. This property is particularly advantageous for functional foods and nutraceuticals intended for extended storage periods.^13^ Recent studies have demonstrated the efficacy of **Hbf** in protecting against acute lung injuries induced by OS and inflammation, primarily through the activation of antioxidative enzymes and pathways, such as AMPK2/Nrf2.^14^

Despite extensive research, several gaps remain in the understanding of the synergistic effects and structural–function relationships of flavonoids. Standardized *in vitro* assays, such as DPPH and FRAP assays, provide valuable quantitative insights into their radical scavenging and reducing power.^15^ This study aimed to characterize the structural and functional properties of **Gspt**, **Gsp**, and **Hbf**, compare their efficacies in diverse OS models, and explore their possible applications in food systems and nutraceuticals. By addressing these objectives, this study seeks to enhance the understanding of natural antioxidants, contributing to innovations in health–oriented and industrial solutions to OS.

## 2 Materials and Methods

### 2.1 Sample Preparation

**Gspt**, **Gsp**, and **Hbf** were obtained from commercial sources (Extrasynthese, France). Stock solutions of each compound were prepared at concentrations of 50, 100, 150, 200, 250 μM/mL in a 1% solution of DMSO in MeOH and stored at – 20 °C until further use. Working solutions were prepared immediately prior to analysis by diluting the stock solutions with methanol or appropriate buffers. To ensure precision, all dilutions were prepared under standardized laboratory conditions and the stock solutions were evaluated for stability prior to experimentation.

### 2.2 DPPH Radical Scavenging Assay

The DPPH assay was conducted to evaluate the free radical scavenging activity of the flavonoids based on the method described by Brand–Williams et al. (1995) and modified for microplate analysis.^16^ A 0.1 mM solution of DPPH in methanol was freshly prepared and protected from light. Test samples (50 μL) at varying concentrations (50, 100, 150, 200, and 250 μM/mL) were added to 150 μL of DPPH solution in a 96–well microplate. The reaction mixtures were incubated in the dark at room temperature for 30 min to ensure consistent interactions between DPPH radicals and the test compounds. Absorbance was measured at 517 nm using a microplate reader. Methanol was used as the blank and DPPH solution without the test compound was used as the control. Trolox was the standard for this study and results were given as Trolox equivalents (TEAC, mM/g). Additionally, the IC_50_ value for DPPH was calculated.

### 2.3 Ferric Reducing Antioxidant Power (FRAP) Assay

The FRAP assay was performed to determine the reducing power of the flavonoids following the procedure established by Benzie and Strain (1996).^17^ This method measures the ability of antioxidants to reduce ferric ions (Fe^3^^+^) to ferrous ions (Fe^2^^+^) under acidic conditions. The FRAP reagent was freshly prepared by mixing 300 mM acetate buffer (pH 3.6), 10 mM TPTZ solution in 40 mM HCl, and 20 mM FeCl_3_ at a ratio of 10:1:1. Test samples (50 μL) at various concentrations (50, 100, 150, 200, and 250 μg/mL) were combined with 150 μL of FRAP reagent in a 96–well microplate. The reaction mixtures were subsequently incubated at 37 °C for 30 min. Absorbance was measured at 593 nm using a microplate reader. Trolox was used as the standard and the results were expressed in terms of Trolox equivalents (TEAC, mM/g). All reagents and standards were freshly prepared and the assay was conducted under uniform conditions to minimize variability.

### 2.4 Statistical Analysis

All experiments were performed in triplicate, and the results are presented as the mean ± standard deviation. Statistical analyses were performed using one–way analysis of variance (ANOVA) followed by Tukey’s post hoc test to determine significant differences between groups. The level of significance was set at *P* < 0.05. Additionally, the IC_50_ values for the DPPH assay were calculated using nonlinear regression analysis, providing a quantitative measure of the half–maximal inhibitory concentration.

### 2.5 Computational Methods

The representative conformer of each substance was generated with CREST program.^18,19^ Quantum–mechanicals computations were carried out using Gaussian16 (rev. C.01)^20^ software package. The geometries were optimized using M05 ^21^/6–31+G­(d)^22–25^ level of theory, followed by the single–point and frequency computations with 6–311+G­(d,p)^23,26^ basis set instead. The choice of functional was dictated its parametrization for metal–involving systems and those without it, hence fitting to both studies on TPTZ–involved studies and DPPH. Additionally, Minnesota functionals are already established through multiple studies to correctly mirror experimental data by means of reactions thermochemistry and kinetics. To account for the solvation effect, water and methanol, corresponding to FRAP and DPPH–related environments, respectively, were simulated with solvation model based on density.^27^

The antioxidative activity was assessed for the species present at the non–negligible molar fractions at pH of the assays. Given lack of the experimental p*K*_a_ values at the time of writing the manuscript, they have been established in water following validated fitted parameters^28^ and converted to methanol,^29^ following approaches tailored specifically for polyphenolic compounds.

The examined mechanisms of actions encompassed formal hydrogen atom transfer (*f*–HAT, for DPPH) and single electron transfer (SET, for DPPH and FRAP) in accordance to QM–ORSA protocol.^30,30–32^ Although post–SET DPPH^–^ intermediate is capable to undergo proton–transfer to form DPPH_2_,^33^ the assay itself measures the decay of purple color originating from DPPH, allowing us to disregard the process which, *nota bene*, is expected to be spontaneous with a great reaction rate. The propensity of the reactions was determined by means of thermochemistry, and kinetic constant for exergonic processes were further examined with tunnelling–corrected transition state theory (as for *f*–HAT), considering 1 M standard state and Marcus theory for electron transfer reactions.

## 3 Results

### 3.1 Comparison of Antioxidative Activity via DPPH and FRAP Assays

The antioxidant properties of **Gsp**, **Gspt**, and **Hbf** were evaluated utilizing DPPH and FRAP assays. These assays assess distinct antioxidant mechanisms, with DPPH measuring hydrogen atom transfer, and FRAP evaluating electron donation.

#### 3.1.1 DPPH Assay

In the DPPH assay (Figure 2), **Gspt** exhibited the highest radical scavenging activity among the three flavonoids, as evidenced by its Trolox Equivalent Antioxidant Capacity (TEAC) value of 111.53 mM/g at the maximum tested concentration (250 μM). **Gsp** and **Hbf** displayed significantly lower TEAC values of 41.68 mM/g and 39.99 mM/g, respectively, under identical conditions. These results indicate the superior capacity of **Gspt** to neutralize DPPH radicals, presumably due to the presence of six hydroxyl groups in its structure, which provide additional sites for hydrogen atom donation. The IC_50_ values derived from the DPPH assay further corroborated the superior activity of **Gspt**. **Gspt** demonstrated the lowest IC_5__0_ value (95.35 mM), indicating high efficiency in scavenging free radicals, even at lower concentrations. In contrast, **Gsp** and **Hbf** exhibited higher IC_5__0_ values of 63.10 mM and 61.31 mM, respectively, reflecting their moderate radical scavenging capabilities. Table 1 presents the IC_50_ values (μM) for **Gspt**, **Gsp**, and **Hbf**, as determined in the DPPH assay. These data suggest that structural features such as glucosyl and glucuronyl substitutions in **Gsp** and **Hbf** may marginally impede their capacity to donate hydrogen atoms. This limitation could be attributed to steric hindrance or the reduced availability of free hydroxyl groups.

**Figure 2:**
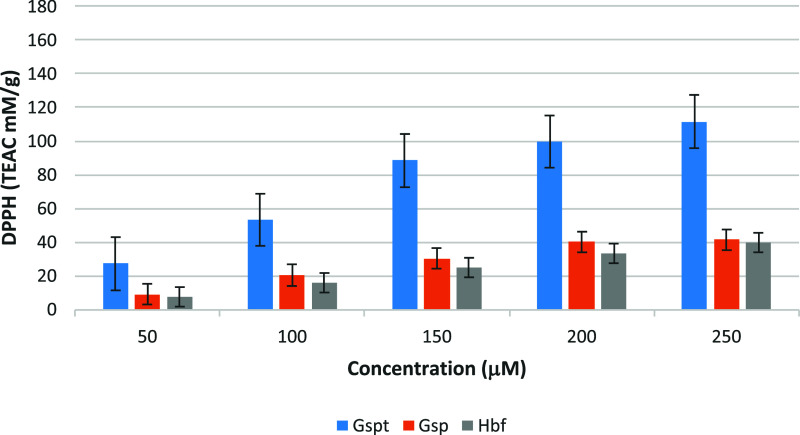
Bar chart comparing antioxidant activity in DPPH assay.

**Table 1:** IC_50_ Values (μM) for **Gspt**, **Gsp**, and **Hbf** Determined in the DPPH Assay

compound	IC_50_ (μM)
Gspt	95.35
Gsp	63.10
Hbf	61.31

#### 3.1.2 FRAP Assay

The FRAP assay (Figure 3) demonstrated a distinct trend when compared to the DPPH results, emphasizing the differential capacity of the tested compounds to donate electrons and reduce ferric ions (Fe^3^^+^) to ferrous ions (Fe^2^^+^). Among the three flavonoids, **Gspt** exhibited the highest reducing power, with a TEAC value of 155.24 mM/g at 200 μM, followed by **Gsp** with 126.28 mM/g, and **Hbf** with 94.67 mM/g. Although **Gspt** showed the highest mean reducing power, the differences among the three flavonoids were not statistically significant at all concentrations The pronounced performance of **Gspt** may be attributed to its structural richness in hydroxyl groups, which facilitates both hydrogen atom transfer and electron transfer mechanisms. Conversely, the slightly lower FRAP values for **Gsp** and **Hbf** might reflect the modulating influence of the glucosyl and glucuronyl moieties, which can affect electron delocalization and reactivity. This contrast between the assays underscores the multifaceted nature of antioxidant behavior and the critical role of specific structural features in shaping redox-related bioactivity.

**Figure 3:**
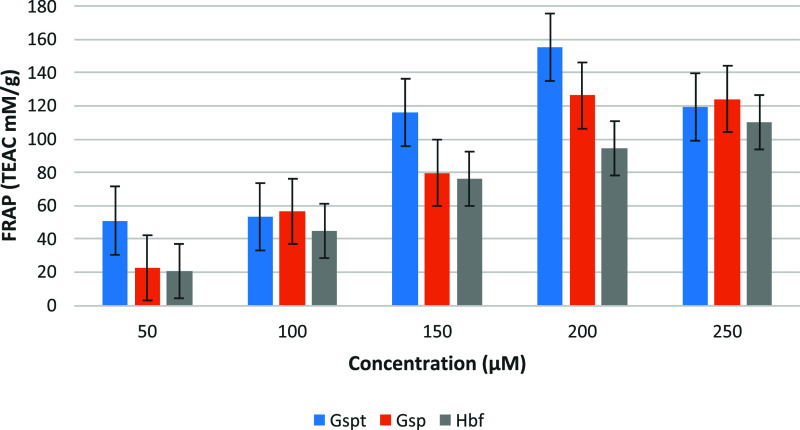
Bar chart comparing antioxidant activity in FRAP assay.

#### 3.1.3 Statistical Analysis

Statistical evaluation using one–way ANOVA confirmed significant differences in antioxidative activity among the three compounds in both assays (*p* < 0.05). Post hoc analyses revealed that the DPPH activity of **Gspt** was significantly higher than those of **Gsp** and **Hbf** (*p* < 0.05). This finding demonstrates **Gspt**’s superior radical scavenging capacity. However, in the FRAP assay, the differences among the three flavonoids were not statistically significant, indicating that **Gsp**, **Gspt**, and **Hbf** exhibited comparable electron–donating capacities under the experimental conditions tested. The statistical outcomes underscore the importance of assay selection when evaluating antioxidative activity, as different methods may yield contrasting results depending on the structural properties and antioxidative mechanisms of the compounds under investigation.

### 3.2 Computational Results

#### 3.2.1 Acid–Base Equilibria

The acid dissociation constants for the studied compounds were determined in water and methanol and are presented in Table 2. The deprotonation sequences for each compound were identified as follows:

a) For **Gspt**: C_7_O–H → C_3′_O–H → C_3_O–H → C_5_O–H → C_4’_O–H → C_8_O–H
b) For **Gsp**: C_7_O–H → C_4’_O–H → C_3_O–H → C_5_O–H → C_3′_O–H
c) For **Hbf**: COO–H → C_4’_O–H → C_7_O–H → C_3_O–H → C_5_O–H → C_3′_O–H

**Table 2:** Dissociation Constants of Each Deprotonation Step

compound	p*K*_a1_	p*K*_a2_	p*K*_a3_	p*K*_a4_	p*K*_a5_	p*K*_a6_
Water						
Gspt	7.16	8.18	10.43	12.37	13.05	14.19
Gsp	7.32	7.82	10.62	12.17	13.41	
Hbf	3.28	7.78	9.01	11.01	12.77	13.24
Methanol						
Gspt	11.27	12.37	14.81	16.91	17.65	18.89
Gsp	11.44	11.99	15.02	16.70	18.04	
Hbf	7.06	11.94	13.28	15.44	17.35	17.86

As anticipated, the carboxylic acid group in **Hbf** exhibits the highest propensity for deprotonation, a characteristic feature of its structure. Among flavonoids, the C_7_ position is frequently the next most acidic site, consistent with the observed sequences. Notably, the second deprotonation step typically involves the B–ring. For **Gspt**, this occurs at C_3′_, whereas for **Gsp** and **Hbf**, it occurs at C_4’_. This variation suggests that the presence of a sugar unit in **Gspt**, although not directly disrupting the delocalized electron system, influences the dissociation site through intramolecular interactions between its hydroxyl groups and those in close proximity.

Analysis of the p*K*_a_ values indicates that, for **Gspt** and **Gsp**, the neutral form predominantly drives activity across all assay conditions. In contrast, **Hbf** exhibits a distinct behavior influenced by its ionization state. In the DPPH assay (pH ∼ 8.22), the molar fractions are 6.51% of the neutral and 93.49% of the anionic form, underscoring the dominant role of the latter. Conversely, in the FRAP assay (pH ∼ 2.56), the molar fractions shift to 84.01% for the neutral and 15.99% for the anion, indicating a reduced yet still significant contribution from the anionic form. This situation is particularly relevant because anionic species, with their enhanced electron density, are more likely to participate in the electron transfer mechanisms, facilitating antioxidant activity.

#### 3.2.2 DPPH Assay

The Gibbs free energies of the reactions between the studied substances and DPPH are presented in a bar chart (Figure 4). Among the hydroxyl groups common to all substances, the highest average ΔG is typically associated with position C_5_, while the lowest is linked to either C_3_ or C_4’_. More specifically, the COOH group of **Hbf** exhibits the highest ΔG, rendering it the least prone to the *f*–HAT mechanism, whereas the hydroxyl group at position C_8_ of **Gspt** displays the lowest ΔG, marking it as the most active site.

**Figure 4:**
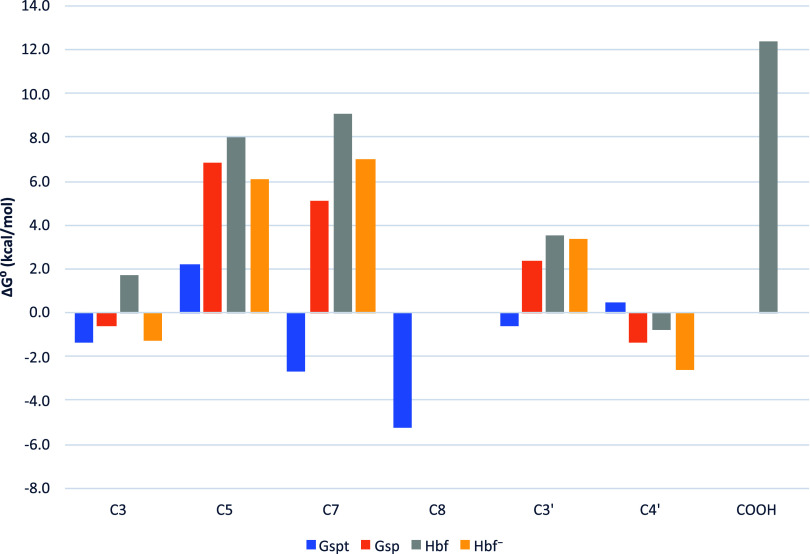
Bar chart comparing Gibbs free energies in DPPH assay.

**Gspt**, characterized by the largest number of hydroxyl groups and lower ΔG values for most positions (except C_4’_), emerges as the most reactive compound, a finding consistent with experimental observations; however, without kinetic calculations, the underlying basis of this activity remains uncertain. In contrast, **Hbf**, the only compound featuring a carboxyl group, may exhibit activity through both neutral and ionized forms, though its hydroxyl groups are associated with the highest ΔG values. Furthermore, the dissociation of the carboxyl group within the sugar moiety enhances reactivity by lowering ΔG values, making reactions more feasible.

The reaction rates estimated for the exergonic sites are tabulated in Table 3. The lowest activation energies are linked to the SET mechanism, with the lowest activation energy associated with **Gspt** and the highest with **Gsp**. Despite the presence of multiple potential reaction sites across these compounds, only a specific subset remains active and contributes to the actually observed reaction rate. Specifically, these active sites are C8 of **Gspt** (*k*_i_ = 6.10 × 10 ° M^–1^ s^–1^) and C_3_ of **Hbf** ^**–**^ (*k*_i_ = 3.17 × 10^1^ M^–1^ s^–1^). All other sites have individual kinetic constants below that of the SET mechanism, suggesting that electron transfer is the predominating path of activity for **Gsp** and **Hbf**. Nonetheless, given the molar fraction of **Hbf** species, it appears that the overall activity stems solely from the anionic form.

**Table 3:** Activation Energies (Δ*G* ^≠^, in kcal mol^–1^), Individual (*k*_i_, in M^–1^ s^–1^), and Molar Fraction-Corrected Total Reaction Rates (*k*_DPPH_, in M^–1^ s^–1^) of Reaction between Studies Species and DPPH Radical

compound	position	Δ*G* ^≠*^	*k*_i_	*k*_total_	*k*_DPPH_
Gspt	C_3_	24.4	5.06 × 10^–3^	3.58 × 10^2^	
	C_7_	21.3	4.92 × 10^–3^		
	C_8_	19.9	6.10 × 10^0^		
	C_3_′	24.7	5.05 × 10^–6^		
	SET	14.0	3.52 × 10^2^		
Gsp	C_3_	26.7	3.88 × 10^–7^	1.97 × 10^–1^	
	C_4_′	24.3	2.07 × 10^–5^		
	SET	18.4	1.97 × 10^–1^		
Hbf	C_4_′	26.1	6.84 × 10^–7^	1.02 × 10^0^	2.26 × 10^1^
	SET	17.1	1.91 × 10^0^		
Hbf^–^	C_3_	24.5	3.17 × 10^1^	2.16 × 10^1^	
	C_4_′	25.3	3.70 × 10^–6^		
	SET	15.2	4.62 × 10^1^		

#### 3.2.3 FRAP Assay

Following Marcus theory, the thermochemistry and kinetics of the single electron transfer process were assessed. The reliability of this approach was previously ascertained by comparing the experimentally measured rate of Cu­(II)-to-Cu­(I) reduction by O_2_^•–^ and Asc^–^ with computational outputs.^34–36^

The results, presented in Table 4, indicate that **Gspt** is the most reactive compound, while **Gsp** is the least reactive. The differences in reactivity between the **Hbf** species are marginal, with reaction rates exceeding 10^9^ M^–1^ s^–1^ in both cases. Regardless of the overall reactivity hierarchy, all studied substances are predicted to effectively reduce the complex of TPTZ-bound Fe­(III).

**Table 4:** Electronic (ΔE, in kcal mol^–1^), Gibbs (Δ*G*, in kcal mol^–1^), and Activation (Δ*G* ^≠^, in kcal mol^–1^) Energies, alongside Individual (*k*_i_, in M^–1^ s^–1^) and Molar-Fraction-Corrected Total Reaction Rates (*k*_FRAP_, in M^–1^ s^–1^) for the Reactions between the Studied Species and the FRAP Reagent

compound	Δ*E*	Δ*G*	Δ*G*‡	*k*_i_	*k*_FRAP_
Gspt	7.4	0.3	1.9	3.71 × 10^9^	
Gsp	10.5	5.0	5.0	9.75 × 10^8^	
Hbf	9.8	4.1	4.2	6.78 × 10^8^	1.74 × 10^9^
Hbf^–^	9.7	2.9	3.4	1.06 × 10^9^	

## 4 Discussion

In this study, we investigated the antioxidative properties of **Gsp**, **Gspt**, and **Hbf** utilizing DPPH and FRAP assays. The results not only revealed differences in antioxidative activities but also emphasized the significance of chemical structures in influencing these activities. These findings, when contextualized within the broader body of research, provide insights into their mechanisms of action and potential applications in the health and industrial sectors.

### 4.1 DPPH Assay

The DPPH assay results demonstrated that **Gspt** exhibited the highest radical scavenging activity among the three tested flavonoids. Its Trolox Equivalent Antioxidant Capacity (TEAC) value of 111.53 mM/g at 250 μM underscores its superior ability to neutralize DPPH radicals. This is further substantiated by its IC_50_ value of 95.35 mM, which indicates its efficacy, even at lower concentrations. The robust performance of **Gspt** can be attributed to the presence of six hydroxyl groups in its structure, which facilitate effective hydrogen atom donation and radical stabilization. Quantum mechanical computations confirm this trend and reveals that, although four exergonic hydrogen atom transfer routes are possible, the primary scavenging potential stems from the single electron transfer mechanism, resulting in the formation of the DPPH^–^ species. This is evidenced by visibly higher rate constants for SET compared to *f*–HAT. The experimental observations align with the results of,^37^ who reported an IC_50_ value of 6.92 mM for **Gspt**, rendering it more effective than the synthetic antioxidant butylated hydroxyanisole (BHA), which had an IC_50_ of 17.34 Mm.

In comparison, **Gsp** displayed moderate radical scavenging activity, with a TEAC value of 41.68 mM/g and an IC_50_ value of 63.10 mM. These results are consistent with previous studies, such as those by,^38^ who observed 95.21% inhibition of DPPH radicals at a concentration of 100 μM, and,^39^ who found an 88.52% inhibition at 100 μg/mL with an IC_50_ of 31 μg/mL. **Gsp**’s performance is likely due to a glucosyl substitution at the C_8_ position, which, while enhancing solubility, sterically hinders hydrogen atom donation. This modification also indirectly reduces the reactivity of the entire system. Computational analysis indicated a decreased propensity for both mechanisms, reflected in lower kinetic constants for these processes.

Although the computational modeling predicts that **Hbf** should outperform **Gsp**, experimental results from the DPPH assay reveal that **Hbf** is the least active, exhibiting a TEAC value of 39.99 mM/g and an IC_50_ value of 61.31 mM. The differences in activity between **Hbf** and **Gsp** are slight, with a TEAC difference of 1.68 mM/g and in IC_50_ difference of 1.79 mM, and both compounds are significantly less active than **Gspt**. As illustrated in Figure 2, increasing the concentration from 200 to 250 mM leads to a more noticeable rise in **Hbf**’s activity compared to **Gsp**’s. This indicates that at higher concentrations, the intrinsic properties of **Hbf** prevail — the reduced effectiveness of **Hbf** can be attributed to its bulky glucuronyl group, which, like the glucosyl group in **Gsp**, restricts access to surrounding hydroxyl groups critical for radical scavenging. Furthermore, under the experimental conditions, **Hbf** exists in both neutral and anionic forms, potentially altering the reaction kinetics. This dual nature may introduce interactions—such as dispersion forces between anions—or effects from the carboxyl group itself, both of which could influence the observed activity.

### 4.2 FRAP Assay

The FRAP assay demonstrated a distinct antioxidative profile in comparison to the DPPH results, highlighting the diverse electron-donating capacities of the flavonoids under investigation. Among the compounds, **Gspt** exhibited the highest reducing power, with a TEAC value of 155.24 mM/g at 200 μM, followed by **Gsp** (126.28 mM/g) and **Hbf** (94.67 mM/g). These findings suggest that **Gspt** is not only highly effective in radical scavenging, as evidenced by the DPPH assay, but also possesses a superior capacity for electron donation, thereby effectively participating in single-electron transfer mechanisms.

Previous studies, such as those conducted by^39^ and,^38^ have highlighted the significant reducing activity of **Gsp**, which may be attributed to its enhanced solubility and favorable electron transfer properties. However, the current data indicate that while **Gsp** is effective, it is surpassed by **Gspt** under the tested conditions. The same observations can be drawn from the computational studies, where rate constants was determined to be equal 9.75 × 10^8^ M^–1^ s^–1^ and 3.71 × 10^9^ M^–1^ s^–1^, respectively. This variation may be due to differences in molecular structure; specifically, the multiple hydroxyl groups in **Gspt** likely enhance its redox flexibility, thereby enabling a superior ferric-reducing capacity. Although the FRAP value of **Hbf** is lower than those of **Gspt** and **Gsp**, it still demonstrates a meaningful electron-donating ability, potentially facilitated by its glucuronyl moiety. This observation is consistent with hypotheses suggesting that structural modifications, such as glycosylation, can differentially modulate antioxidative behavior, sometimes enhancing electron transfer while limiting hydrogen atom donation.

While^37^ reported a lower FRAP value for **Gspt** (3.72 mM/L) compared to gallic acid (4.49 mM/L), such discrepancies may arise from methodological variations, including concentration ranges, solvent systems, or reference standards. Therefore, direct comparisons across studies should be approached with caution, and further investigations under standardized conditions are warranted. Collectively, the findings from the FRAP assay confirmed that **Gspt** exhibits the most potent reducing capacity among the studied flavonoids, demonstrating versatility across both electron transfer and hydrogen atom transfer pathways. This dual mechanism suggests that **Gspt** is the most effective antioxidant candidate within this experimental framework.

### 4.3 Comparative Analysis

These results elucidate the role of structural variations in shaping the antioxidative properties of flavonoids. **Gspt’s** outstanding DPPH and FRAP activity can be attributed to its multiple hydroxyl groups, which facilitate both hydrogen atom transfer and electron donation. In contrast, **Gsp** and **Hbf**, while exhibiting lower radical scavenging ability, still demonstrated meaningful reducing power in the FRAP assay, likely due to glucosyl and glucuronyl substitutions that enhance solubility and electron mobility.

The observed differences in performance between the DPPH and FRAP assays highlight the impact of flavonoid substitution patterns on antioxidant mechanisms. The consistently high performance of **Gspt** in both assays indicates a structure well-suited for redox activity, corroborating previous studies (e.g.,^37^) that have reported its superior radical scavenging capabilities compared to synthetic antioxidants. **Gsp’s** strong performance in the FRAP assay is consistent with the findings of^39^ and,^38^ who associated glycosylation with enhanced aqueous solubility and electron transfer efficiency. **Hbf**, which contains a glucuronyl group, exhibited a similar pattern, supporting the perspective advanced by^14^ that such substitutions enhance stability and reducing capacity. These insights suggest that **Gspt** may be an optimal candidate for therapeutic interventions targeting ROS-related pathologies, such as neurodegenerative and inflammatory conditions. Concurrently, the robust reducing power of **Gsp** positions it as a promising antioxidant for applications in food stabilization, particularly where the prevention of oxidative spoilage is critical. **Hbf’s** balanced antioxidative profile supports its use in functional foods and nutraceuticals aimed at long-term shelf stability. Overall, these findings underscore the necessity of tailoring the application of flavonoids based on the predominant antioxidative mechanism: hydrogen atom transfer versus electron donation

**Gspt** demonstrated the most potent activity in the DPPH assay, which can be attributed to its structural abundance of hydroxyl groups, facilitating effective radical scavenging. This finding aligns with the research of,^37^ who established **Gspt**’s superior antioxidant activity in comparison to synthetic standards such as BHA. Conversely, **Gsp** exhibited moderate DPPH activity but showed strong performance in the FRAP assay, consistent with the results reported by^39^ and,^38^ who observed that glycosylation can enhance solubility and electron transfer efficiency. Although **Hbf** displayed limited radical scavenging ability, it demonstrated significant FRAP activity, indicating that glucuronylation enhances electron donation capacity. This pattern is in agreement with the findings of,^14^ who highlighted the dual role of glucuronyl substitutions in augmenting compound stability and redox potential.

Given its remarkable performance in DPPH and FRAP assays, **Gspt** has emerged as a promising candidate for therapeutic applications, particularly in the management of oxidative stress-related disorders, such as neurodegenerative and inflammatory conditions. The strong reducing power of **Gsp** suggests its potential utility in preserving the quality of food products susceptible to oxidative spoilage. Meanwhile, the balanced antioxidant profile of **Hbf** is suitable for incorporation into functional foods and nutraceuticals that require stability over extended storage periods. These distinct antioxidative profiles underscore the importance of tailoring flavonoid application strategies according to their predominant redox mechanisms.

These findings build upon previous reports of **Gspt**’s activity (e.g.,;^37^ TEAC and IC_50_ not reported), now offering for the first time a comprehensive comparative kinetic and thermodynamic quantification. For **Gsp**, while its antioxidant efficacy was previously documented (;^39^ IC_50_ = 31 μg/mL), our study provides the inaugural evaluation across both **HAT** and **SET** mechanisms, supported by computational and p*K*_a_ modeling. Finally, although **Hbf**’s antioxidant capacity was suggested for its anti-inflammatory potential, it has not been previously quantified in FRAP/DPPH assays.^14^ This study is the first to establish its dual redox behavior both experimentally and computationally.

### 4.4 Research Gaps and Future Directions

Despite significant advancements in understanding the antioxidative properties of **Gspt**, **Gsp**, and **Hbf**, several research gaps persist. First, although the individual properties of these flavonoids are well documented, the synergistic effects of their combinations remain unexplored. Investigating their potential interactions could elucidate enhanced antioxidative mechanisms and multifunctional applications in both therapeutic and food systems.

The current understanding of the structure–activity relationships of these compounds is also incomplete. Although this study highlights the influence of hydroxyl, glucosyl, and glucuronyl groups on antioxidative activity, more comprehensive computational and experimental studies are necessary to optimize derivatives with superior properties. Additionally, hibifolin remains underexplored compared with gossypetin and gossypin. Its unique glucuronyl group warrants further investigation to elucidate its impact on antioxidant mechanisms, enzymatic interactions, and applications in complex biological and food matrices.

Application–specific studies are critical areas that require further attention. While the general applications of these flavonoids in antioxidative roles are promising, their stability, bioavailability, and performance under real–world conditions, such as high–temperature food processing or pharmaceutical delivery systems, need to be systematically evaluated. Moreover, the role of these compounds in modulating oxidative enzymes and their long–term stability in functional foods and nutraceuticals remains largely unknown. However, a significant gap exists between *in vivo* and clinical studies. Most current research, including this study, focuses on in vitro assessments, which, while informative, do not account for bioefficacy, metabolism, and safety in living systems. Expanding the research to include in vivo models and clinical trials is essential to bridge this gap.

In addition to antioxidative properties, flavonoids, such as **Gspt**, **Gsp**, and **Hbf**, may also possess other biological activities, such as anti–inflammatory or anti–cancer effects, which are underexplored. Investigating these additional roles could broaden their potential applications and increase their value in therapeutic and industrial applications.

Finally, there is a need for research on industrial scalability and cost–effectiveness. Laboratory findings often do not translate readily into commercial application. Studies focusing on production scalability, economic viability, and integration into industrial processes are vital for transforming flavonoids from experimental compounds into widely usable products. Addressing these gaps through comprehensive and targeted research will pave the way for their innovative applications in health, nutraceuticals, and food science.

## 5 Conclusions

This investigation elucidates the importance of structural variations in determining the antioxidative efficacy of **Gsp**, **Gspt**, and **Hbf**. **Gspt** demonstrated the most potent radical scavenging activity. **Gsp** demonstrated robust reducing power, though differences among flavonoids in the FRAP assay were not statistically significant. These findings contribute valuable insights into the structure–activity relationships of flavonoids, facilitating targeted applications in health and industry. Subsequent research should explore the synergistic potential of combining these flavonoids to optimize their antioxidant effects.

## Supplementary Material



## Notes

Details on p*K*_a_ and molar fraction estimation (PDF)
