# Supplementary material for: Aglycone, Glycoside, or Glucuronide? Experimental and Mechanistic Insights into the Antioxidative Potential of Gossypetin, Gossypin, and Hibifolin
Source: J Phys Chem B. 2025 Jul 16;129(29):7593–601. doi: 10.1021/acs.jpcb.5c03338 (PMC12302068; doi:10.1021/acs.jpcb.5c03338)
Supplement: Supplementary file 1 [file jp5c03338_si_001.pdf]

Aglycone, Glycoside or Glucuronide? Experimental and Mechanistic Insights into the Antioxidative Potential of Gossypetin, Gossypin and Hibifolin

Maciej Spiegel[a]*, Adam Kowalczyk[b]

[a] Department of Organic Chemistry and Pharmaceutical Technology, Faculty of Pharmacy, Wroclaw Medical University, Borowska 211A, Wroclaw, Poland

[b]Department of Pharmacognosy and Herbal Medicine, Faculty of Pharmacy, Wroclaw Medical University, Borowska 211A, Wroclaw, Poland

E–mail address: maciej.spiegel@umw.edu.pl

1. Acid–Base Equilibria

Following the methodology described in the main text, the dissociation pathways of the studied compounds and their corresponding pK_a values were evaluated in the methanolic medium of the DPPH assay and the aqueous medium of the FRAP assay (**Table S1**). Dissociation begins either at the C_7 hydroxyl group (for gossypetin, **Gspt**, and gossypin, **Gsp**) or the carboxyl group (for hibifolin, **Hbf**)—both patterns are expected—followed by the hydroxyl group on the B-ring. Between the solvents, pK_a values increase in methanol, which is anticipated due to its less polar nature. Simultaneously, pK_a values differ only slightly between **Gspt** and **Gsp**, whereas more pronounced differences are observed for **Hbf**, attributable to the presence of an easily dissociating carboxyl group. At the time of writing this paper, no experimental data were available; thus, the values established here should be adopted. Consequently, molar fractions were determined (**Figures S1–S3**), revealing that in the DPPH assay, the predominant form is neutral for all compounds except **Hbf**, where neutral and anionic forms exist in nearly equal concentrations. In the FRAP assay, this trend persists, but the **Hbf**:**Hbf⁻** ratio is approximately 1:2.

Table S1. Theoretical Deprotonation Constants of the Studied Substances in Water and Methanol.

	pK_{a1}	pK_{a2}	pK_{a3}	pK_{a4}	pK_{a5}	pK_{a6}
Gspt	C_7	$C_{3'}$	C_3	C_5	$C_{4'}$	C_8
methanol	11.27	12.37	14.81	16.91	17.65	18.89
water	7.16	8.18	10.43	12.37	13.05	14.19
Gsp	C7	C4’	C3	C5	C3’	
methanol	11.44	11.99	15.02	16.70	18.04	
water	7.32	7.82	10.62	12.17	13.41	
Hbf	COOH	C4’	C7	C3	C5	C3’
methanol	7.06	11.94	13.28	15.44	17.35	17.86
water	3.28	7.78	9.01	11.01	12.77	13.24

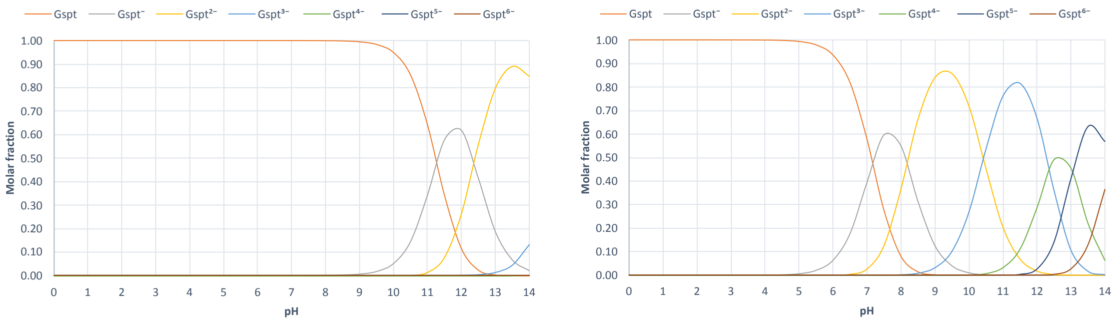

Figure S1. Plot of a **Gspt** Species Molar Fractions as a Function of pH in DPPH (left) and FRAP (right) Conditions.

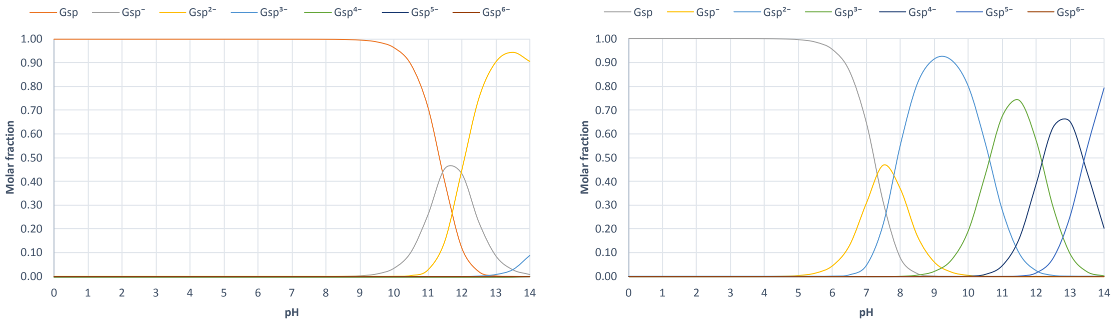

Figure S2. Plot of a **Gsp** Species Molar Fractions as a Function of pH in DPPH (left) and FRAP (right) Conditions.

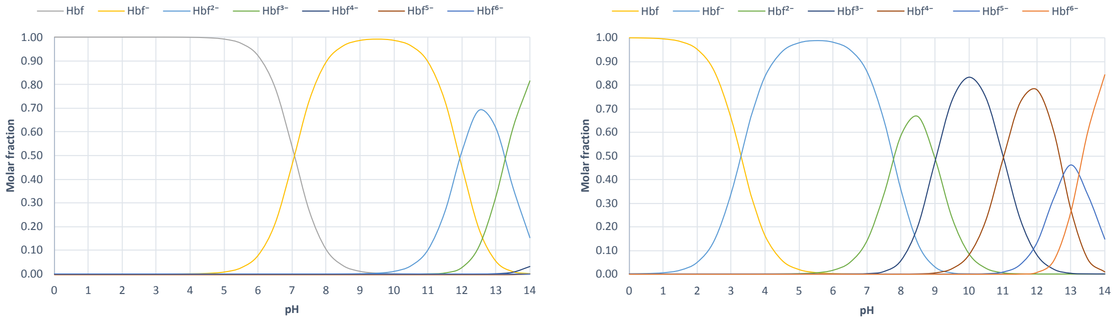

Figure S3. Plot of a **Hbf** Species Molar Fractions as a Function of pH in DPPH (left) and FRAP (right) Conditions.
